# Effects of *Hermetia illucens* Larvae Meal and Astaxanthin as Feed Additives on Health and Production Indices in Weaned Pigs

**DOI:** 10.3390/ani13010163

**Published:** 2022-12-31

**Authors:** Kinga Szczepanik, Iwona Furgał-Dierżuk, Łukasz Gala, Małgorzata Świątkiewicz

**Affiliations:** Department of Animal Nutrition and Feed Science, National Research Institute of Animal Production, Krakowska St. 1, 32-083 Balice, Poland

**Keywords:** insect meal, astaxanthin, pigs, growth performance, biochemical blood indices, hematological blood indices

## Abstract

**Simple Summary:**

Weaning is a stressful period that reduces digestive capacity and increases oxidative stress and disease susceptibility in piglets. Feed additives can protect the piglets’ health status in a natural way. This study aimed to evaluate the effects of full-fat *H. illucens* larvae meal (HI) and astaxanthin (AST) supplementation on the growth performance and health status of weaned pigs. HI contains bioactive substances (chitin, antimicrobial peptides, lauric acid) with immunostimulatory, antimicrobial, and anti-inflammatory properties. Astaxanthin is a carotenoid pigment with strong antioxidant and anti-inflammatory capacities. The results showed that astaxanthin supports the inhibition of oxidative stress. In the experiment lasting from 35 to 70 days of age, 48 weaned pigs (about 8.7 kg body weight) were involved. Both supplements were tested separately or combined in feed mixtures. The 2.5% HI and AST supplementation can reduce the susceptibility of pork fat to oxidation. However, a higher concentration of HI (5%) was not beneficial because of the adverse changes in some of the red cell indices and thus should be combined with the antioxidant AST to improve these indices. Both supplements did not negatively affect the piglets’ productivity.

**Abstract:**

Weaning is a critical period in farming, and therefore, searching for health-promoting feed additives of natural origin is necessary. This study aimed to evaluate the effects of full-fat *H. illucens* larvae meal (HI) and astaxanthin (AST) supplementation on the growth performance and health status of weaned pigs. The experiment was carried out on 48 pigs (8.7 kg) divided into six groups: I—control; II—2.5% HI; III—5% HI; IV—2.5% HI and AST; V—5% HI and AST; VI—AST. The experiment lasted from the 35th to 70th day of age, and animals were fed ad libitum. The results obtained indicate that HI meal and astaxanthin had no effect on feed intake and utilization, weight gain, or organ weight. Additionally, blood parameters remained within the norms. It seems that astaxanthin supports the inhibition of oxidative stress, which became apparent in the case of some red blood cell parameters. The 2.5% HI and AST supplementation can reduce the susceptibility of pork fat to oxidation (lower adipose tissue TBARS). However, 5% HI in feed was not beneficial because of the adverse changes in some red cell indices, and it should be combined with the antioxidant AST to improve these indices.

## 1. Introduction

One of the major problems generating economic losses in pig farming is the weaning period of piglets [1]. There is a very stressful period of the animal’s life, involving separation from the sow, environmental and nutritional changes increasing exposure to pathogens and food antigens [2], and a new group hierarchy. Weaning from the sow disrupts the intestinal integrity of piglets, reduces the digestive capacity of the digestive system, and increases intestinal oxidative stress and disease susceptibility in piglets [3]. One of the key factors affecting piglet health is diet, and an important component of diet is an easily digestible protein with a favorable amino acid composition. Currently, the main protein sources for monogastric animals are post-extraction meals and oil-pressed cakes (e.g., soybean, rapeseed), legumes seeds, animal origin proteins, or algal biomass [4,5,6]. The rapidly growing human population and the increasing demand for meat and products of animal origin have increased the need for protein feed quantities. The search for new protein sources has become necessary because of the scarcity of plant-based feed protein sources due to unfavorable climate changes and the aversion to genetically modified feedstuffs, so the search for new protein sources has become necessary [6]. 

Attention has begun to turn to insect meal, which could provide an additional source of nutritional components [7,8,9]. The innovative protein products obtained from various insect species have already started to be used for salmonid feeds [10,11] as well as pet food [12]. As a result, interest in black soldier fly (*Hermetia illucens*) larvae (HI) as a sustainable protein source for livestock has increased significantly. Insects’ production advantage is that they can be reared at high densities and have a high bioconversion rate [9]. Organic biomass, byproducts, or food waste can be used for their production, which contributes to more efficient management of organic and inorganic nutrient resources, particularly nitrogen and phosphorus recycling [8,13]. There are currently two types of *H. illucens* larvae meal in feedstuffs: defatted and full fat, in which the main difference is the fat and saturated fatty acid content [6]; in the present experiment, a full-fat meal from *H. illucens* larvae was used. The amount of crude protein in the full-fat HI meal was 426 g/kg, crude fat content was 264 g/kg, crude fiber content was 91 g/kg, and ash content was 85 g/kg. Noteworthy is the amino acid composition of black soldier fly larvae meal. The most abundant essential amino acids were leucine (26.2 g/kg), lysine (21.6 g/kg), and phenylalanine + tyrosine (36.2 g/kg). Promising in terms of improving the health status of animals is the presence in insects of bioactive substances such as chitin, antimicrobial peptides, and specific fatty acids (notably lauric acid) with immunostimulatory, antimicrobial, and anti-inflammatory properties [14,15]. These bioactive compounds appear to be useful feed additives to support the growth and health of piglets by stimulating their immune response, which is important when conducting intensive livestock production and limiting therapy, especially with antibiotics. 

The young piglet’s body develops rapidly, which is associated with an accelerated metabolism. This and weaning stress affect the production of significant amounts of free radicals [16]. Reactive oxygen species from the mitochondrial electron transport chain or excessive stimulation of NAD(P)H) cause oxidative stress can be important mediators of damage to cellular structures, including lipids and membranes, proteins, and DNA [17]. Therefore, adding antioxidants to the diet seems beneficial, which can help counteract the negative effects of oxidative stress [18]. As an antioxidant in the present experiment, astaxanthin was used, which is one of the carotenoid pigments with strong antioxidant, anti-inflammatory, and anticancer properties [19]. The antioxidant properties of astaxanthin are 14 times greater than those of vitamin E, 54 times greater than those of β-carotene, and 65 times greater than those of vitamin C [20]. In addition, astaxanthin is believed to protect against apoptosis by regulating mitochondrial proteins [21]. A study by Macedo et al. (2010) [22] found that astaxanthin, by reducing the level of pro-inflammatory cytokines in lipopolysaccharide-stimulated neutrophils, improves the phagocytic capacity of neutrophils and their bactericidal capacity, and reduces the amount of hydrogen peroxide and superoxide anion they produce. 

Given the above, this experiment aimed to study the effects of *Hermetia illucens* larvae meal and astaxanthin as feed additives, with the potential to improve the health status and production indicators of weaned pigs. The blood health indices (biochemical and hematological), growth performance, and meat quality traits were estimated.

## 2. Materials and Methods

### 2.1. Ethical Approval

All procedures included in this study relating to the use of live animals agreed with the First Local Ethics Committee for Experiments with Animals in Cracow, Poland (Resolution No. 420/2020, date 22 July 2020). Throughout the experimental period, the health status of postweaning pigs was regularly monitored by a veterinarian.

### 2.2. Animals and the Layout of the Experiment

The experiment was conducted on forty-eight 35-day-old post-weaning pigs (barrows) weighing about 8.7 kg (±0.2 kg). The barrows were of the Polish Landrace (PL) breed. The pigs were divided into six groups, with eight pigs in each: group I—control, group II—addition of 2.5% *Hermetia illucens* (HI) larvae meal, group III—addition of 5% *H. illucens* larvae meal, group IV—addition of 2.5% *H. illucens* larvae meal and astaxanthin, group V—addition of 5% *H. illucens* larvae meal and astaxanthin, group VI—addition of astaxanthin. The *Hermetia illucens* larvae meal was a full-fat product obtained from commercial sources (HiProMine S.A., Robakowo, Poland). The astaxanthin originated from *Haematoccocus pluvialis* (Podkowa AD 1905 sp. z o.o., Lublin, Poland) and was added in the amount of 0.025 g per 1 kg (25 mg per kg) of feed mixture. All piglets were fed an iso-protein and iso-energetic diet, meeting the requirements according to the Polish standards of pig feeding [23]. The ingredient composition and nutritive value of the diets are shown in Table 1. Basic chemical analyses of feed mixture samples were performed according to standard methods [24].

The experimental fattening lasted 35 days. The pigs were kept in individual pens and received feed and water ad libitum. The animals were individually weighed on the experiment’s first and last day. Daily feed intake and conversion, as well as animal weight gain, were calculated. At the end of the experiment, all pigs were slaughtered. The animals were killed with an approved standard method by simply stunning with a specialized penetrating pin device Blitz (Germany), along with cartridges caliber 9 × 17 mm dedicated to slaughtering pigs. Blood was collected in tubes for biochemical and hematological analysis. Intestine sections, kidneys, stomach, liver, and spleen were collected for weighing. Samples of muscle (*longissimus m.*) and adipose (backfat) tissue were also taken from the area between the last thoracic and first lumbar vertebrae. The dissected intestine sections (duodenum, jejunum, ileum, cecum, and large intestine) were rinsed, weighed, and measured. The pH of the stomach, duodenum, jejunum, ileum, large intestine, and caecum digesta was measured with a HI 99163 pH-meter (Hanna Instruments Inc., Woonsocke, RI, USA), with automatic temperature compensation from −5 to 105 °C and equipped with a pH/T° FC 232 combination electrode.

### 2.3. Blood Analysis

#### 2.3.1. Hematological Parameters

The full blood samples were analyzed using the Vet Mythic 18 automatic hematology analyzer (Orphée C2 Diagnostics, France). The parameters evaluated were total white blood cell (WBC) count, lymphocytes (LYM), monocytes (MON), granulocytes (GRA), red blood cells (RBC) count, hemoglobin content (HGB), hematocrit (HCT), mean corpuscular volume (MCV), mean corpuscular hemoglobin (MCH), red cell distribution width (RDWC), number of platelets (PLT) and their mean volume (MVP), platelet size heterogeneity index (PDW), and plateletcrit (PCT).

#### 2.3.2. Biochemical Parameters

Blood samples for the biochemical parameters were collected in test tubes and centrifuged (3500× *g*, 15 min, 4 °C) to obtain serum samples. The biochemical indices were colorimetrically measured using test Cormay kits (Lublin, Poland) and a BS-180 biochemical automatic analyzer (Shenzhen Mindray Bio-medical Electronics Co. Ltd., Shenzhen, China). The following parameters were determined: total cholesterol (CHOL), high-density lipoprotein (HDL), low-density lipoprotein (LDL), triacylglycerides (TG), lactate dehydrogenase (LDH), alanine aminotransferase (ALT), aspartate aminotransferase (AST), alkaline phosphatase (ALP), glucose (GLU), albumin (ALB), creatinine (CREA), urea (UREA), total protein (TP), calcium (Ca), phosphate (P), magnesium (Mg), iron (Fe).

### 2.4. Meat and Backfat Sample Collection and Analysis

Samples of meat (*longissimus m*.) and adipose tissue (backfat) were taken from the area between the last thoracic and the first lumbar vertebrae. Basic chemical analyses (dry matter, crude protein, crude fat, and crude ash) of meat samples were performed according to standard methods [24]. Thiobarbituric acid reactive substances (TBARS) were analyzed in meat and backfat samples after 3 months of storage at –20 °C, using a modified method proposed by Pikul et al. (1989) [26]. In brief, 10 g of shredded sample was homogenized with 50 mL of 4% perchloric acid with butylated hydroxytoluene. After filtration, 5 mL of the filtrate was mixed with 5 mL of 2-thiobarbituric acid (0.02 M). The solution was heated in a test tube for 1 h, in a boiling water bath, and then cooled under running water for 10 min. The measurement was carried out at 532 nm against a calibration curve containing a blank sample.

### 2.5. Statistical Analysis

Data were analyzed by 2-way ANOVA using Statistica^®^ ver. 13.3 software packages (StatSoft Inc., Tulsa, OK, USA) [27]. The model included two main factors: (1) *Hermetia illucens* larvae meal share (2.5% vs. 5.0%) and (2) the astaxanthin presence in the feed mixture, and their interactions. Each individual piglet served as an experimental unit (n = 8, per group). Before the data analysis, the normality of the data was tested using the Shapiro–Wilk test and histograms were evaluated. Duncan’s test was used to compare differences between averages when the difference was found to be significant (*p* < 0.05).

## 3. Results

### 3.1. Growth Performance

All animals were healthy during the experiment and showed no signs of disease. Indicators of weight gain, feed conversion ratio, average daily gain, feed intake, and parameters collected during dissection are shown in Table 2 and Table 3. There were no statistically significant differences between the groups.

### 3.2. Blood Indices

The effects of insect meal from *Hermetia illucens* larvae administered at different doses and astaxanthin on the biochemical blood indices, as well as the interaction between these factors, are shown in Table 4. Lipid profile was not affected by the HI meal, except HDL (*p* = 0.03) and LDH content (*p* < 0.01), and not by the astaxanthin supplementation in feed. Analyzing the hepatic/pancreas and the renal and osteological profiles, some varied effects of experimental nutritional factors were observed. HI meal lowered the GLU content (*p* < 0.05) when added at 5% in the feed, while the astaxanthin supplementation increased the GLU and ALP contents. However, in the case of ALP as well as ALB content, the interaction was statistically significant: these parameters were higher when astaxanthin was added to the feed mixture together with the HI meal. The 2.5% HI meal supplementation in feed increased the *p*-level (*p* < 0.01) and decreased the CREA level (*p* = 0.02) in piglets’ blood, while 5% HI meal supplementation lowered the Ca level (*p* < 0.01). The Mg content in the blood was not affected by the HI meal addition in feed. The astaxanthin increased CREA, Ca, and Mg levels (*p* < 0.01). The interaction (*p* < 0.01) between both nutritional factors was noticed in the TP amount in the blood, which was the lowest in piglets receiving a feed mixture containing HI meal without astaxanthin.

The results of the hematological analysis of piglet blood are shown in Table 5. Astaxanthin supplementation did not affect white blood cell counts, while the 5% HI meal increased LYM counts (*p* = 0.04). Significant interactions indicate that MON and GRA were affected only when both dietary factors were used together, and the highest amount of MON and GRA was observed in piglets fed a mixture containing 5% HI meal along with AST (*p* = 0.01 and 0.02, respectively). Both HI meal and AST affected red blood cell parameters (*p* < 0.05), but the interaction was significant for HCT and MCV only. The lowest values of these parameters were read for the groups fed 5% *HI* meal supplementation (*p* < 0.01; *p* = 0.02). Analyzing the main factors, a significant increase in the level of RDWC after the addition of AST and 5% HI was noticeable (*p* < 0.01). The number of RBCs increased after the addition of AST (*p* < 0.01) but was not affected by HI meal in the diet. The Fe level was lower in the blood of piglets fed with HI meal (*p* = 0.01) but was about 30% higher after the addition of astaxanthin (*p* < 0.01). HGB level decreased after supplementation with AST (*p* < 0.01) and 5% HI meal (*p* = 0.02) meal. Both AST and 5% HI meal decreased MCH (*p* < 0.01). In the case of platelet parameters, the only effect was observed in PDW when 2.5% *HI* meal was used in the feed mixture, which significantly reduced this value (*p* < 0.01).

### 3.3. Meat and Backfat Analysis

The effects of astaxanthin and H. illucens larvae meal on the basic chemical analysis of meat are shown in Table 6. The highest dry matter of meat was determined in piglets treated with 2.5% HI meal or 2.5% HI meal together with AST (interaction *p* = 0.02). The lowest percentage of ash in meat (calculated in dry matter) was determined in the group treated with 2.5% HI meal (*p* < 0.01) and in groups not treated with AST (*p* = 0.03). The protein and fat content in meat (calculated in dry matter) were not affected by HI meal nor AST supplementation in feed.

The results of measurements of oxidative stability of meat and adipose tissue from pigs fed with a mixture containing *Hermetia illucens* meal or astaxanthin are presented in Table 6. Both HI meal and AST significantly decreased the TBARS in adipose tissue (backfat) after 3 months of frozen storage (*p* < 0.01), and the interaction between these factors resulted *p* < 0.01. In comparison to the control group, the 2.5% HI concentration was more effective than the 5% HI concentration (TBARS decreased by 80% vs. 69%), and the AST was more effective alone or together with 2.5% HI added to the feed mixture (TBARS decreased by about 77%). However, in the case of meat, the HI meal supplementation did not influence the TBARS value, while the AST supplementation increased this parameter (*p* < 0.05).

## 4. Discussion

### 4.1. Growth Performance

The inclusion of *H. illucens* larvae meal in the diet did not adversely affect the growth performance of the piglets involved in this study, and no effect of HI meal was observed on the weight of organs and digestive tract sections of piglets (calculated as % of body weight). In contrast, in the experiment of Yu et al. (2020) [28], piglets fed with a mixture containing 0%, 1%, 2%, or 4% of HI meal showed a linear increase in the pancreas and small intestine in response to this diet supplementation. No negative effects on feed intake, feed conversion ratio, or average daily gain were observed. The fact that the presence of HI meal in the feed did not impair the feed intake of the piglets is a favorable result and confirms that insect-originated feed is palatable to these animals. The interest of piglets and their willingness to eat black fly larvae have also been observed by other authors [29]. Conclusions similar to ours were reached by Biasato et al. (2019) [30], who carried out an experiment on weaned piglets fed defatted *H. illucens* larvae meal. The HI larvae meal was included in increasing amounts (0%, 5%, or 10%) in diets formulated for two feeding phases: I (from day 1 to 23) and II (from day 24 to 61). No significant differences in growth performance were observed, except for average daily feed intake in phase II, which showed a linear response to increasing levels of HI meal. Additionally, no effect was observed on the growth performance of weaned piglets fed diets containing up to 8% full-fat HI meal for 15 days [31]. No differences in piglets’ performance were found also by Driemeyer (2016) [32] when fish meal was partially replaced by HI meal. The researcher fed piglets (10 to 28 days of age) on a four-week phase feeding schedule with a diet containing 3.5% HI meal. There were no significant differences obtained for feed intake and average daily gains of the animals. In contrast, in the study by Chia et al. (2021) [33], an effect of *H. illucens* meal on increased daily weight gain was observed. Carcass weights of pigs fed diets with HI meal as a replacement for a fish meal at 50%, 75%, or 100% (*w*/*w*) were higher than those of pigs fed a control diet. In the groups receiving 50% and 100% insect meal in place of fish meal, final body weight was significantly higher than in the control and 25% insect meal-treated groups. In our experiment, no significant differences in final body weight were observed among groups, and no significant differences in feed conversion ratio (FCR) were shown. In contrast, in the experiment with 50%, 75%, or 100% insect meal, FCR was significantly lower than in the control and 25% insect meal groups [33]. In another study [28], crossbred pigs weighing approximately 76.0 kg were assigned to three groups in which they received increasing levels of *H. illucens* meal (0%, 4%, or 8%). The results showed that the 4% HI diet significantly increased the final body weight and average daily weight gain of the pigs and decreased the feed to gain ratio compared to the 0% and 8% HI diets. There were no differences in average daily feed intake among all three groups. One study [34] was conducted for 40 days to investigate the effect of increasing levels of HI larvae oil supplementation on the growth performance of newly weaned pigs (at 21 days of age) reared in a three-phase feeding program. It was found that supplementation with 0%, 2%, 4%, or 6% of insect oil linearly increased (*p* < 0.05) body weight on days 14, 21, 25, 33, and 40, but did not affect the feed intake throughout the whole experiment. However, daily weight gains and feed conversion ratios were linearly improved only in the first rearing period from 0 to 14 days of the experiment. When the weaned piglets received a feed mixture containing 5%, 10%, or 20% of HI meal [35], no significant linear effect was observed in weight gain and feed efficiency. Looking at the nutritional factor, which was an insect product from *Hermetia illucens*, it is conceivable that the variety of results observed in the studies cited above may be due to both the type of product (meal, oil) and the period in which the pigs were included the experiment. This statement is consistent with the observation of a linear improvement in both ADG and FCR when the supplement of HI meal in feed increased from 0%, 1%, 2%, to 4% in the two first weeks post-weaning, whereas no differences were found for a four-week feeding period [36].

The significant effect of astaxanthin supplementation in the amount of 25 mg per 1 kg of feed on the growth performance of weaned piglets was not observed in the present experiment. Similarly [37], the addition of astaxanthin to the pigs’ diet (1.5 or 3 mg per kg of feed) did not affect the average daily gain, average daily feed intake, or feed conversion ratio. When analyzing the nutritional factor astaxanthin, it is important to keep in mind the small number of papers describing the effect of AST supplementation on production performance in pigs. Therefore, the discussion must be expanded to include other monogastric species. Ao and Kim (2019) [38] experimented on Peking ducks that were fed astaxanthin originating from *Phaffia rhodozyma*. A total of 1440 female 1-day-old Peking ducks (approximately 52 g) were divided into three groups: control group—0 mg AST/kg diet, group I—3458 mg AST/kg diet, and group II—6915 mg AST/kg diet. It was found that on days 22 to 42, the inclusion of AST increased weight gain and decreased the feed to gain ratio. Throughout the experiment, weight gain and final body weight were greater in the AST treatment compared to the control group. AST supplementation in the amount of 25 mg per 1 kg of feed, as in the present experiment, did not affect organ weights. In an experiment by Jeong and Kim (2014) [39], 1-day-old male chicken broilers were used to test the effect of AST originated from *P. rhodozyma* on animal rearing rates. The birds received a supplement of 0, 2.3, or 4.6 mg AST/kg feed. The inclusion of AST improved weight gain at finishing and throughout the experimental period and reduced the feed conversion ratio at finishing. Thus, it was suggested that AST supplementation may improve weight gain and reduce the feed conversion ratio. Lei and Kim (2014) [40] evaluated the effects of AST derived from *Phaffia rhodozyma* on the performance and nutrient digestibility of finishing pigs. For this purpose, crossbred pigs (initial body weight of about 58 kg) were treated with 0%, 0.1%, or 0.2% supplementation of *P. rhodozyma*, in which AST content was 2.305 mg/kg after fermentation and freeze-drying. The results showed that the addition of *P. rhodozyma* improved feed efficiency and dry matter digestibility. Evaluating the effect of increasing dietary astaxanthin (0, 5, 10, or 20 mg/kg) on late-finishing pig performance [41], it was found that the growth performance of pigs fed the astaxanthin did not differ from pigs fed a control diet. In our study, astaxanthin was derived from *Haematococcus pluvialis*, which could explain the lack of significant changes between groups compared to work where the source of AST originated from *Phaffia* yeast. However, as shown in studies [42,43], a diet with 133 or 266 mg/kg of *Haematococcus pluvialis* algae caused faster weight gain and significantly higher breast muscle mass, and higher feed efficiency in broiler chickens. Perhaps the AST dose used in this study was too low to be effective in the productivity indicators.

### 4.2. Blood Indices

Although statistically significant differences were observed between groups, the hematological and biochemical blood indices were within the physiological norms [44], indicating that the use of HI insect meal and astaxanthin did not adversely affect the health status of the weaned piglets. When studying the interaction between *H. illucens* meal and astaxanthin on hematological blood indices, attention should be paid to the effects of these factors both together and separately, as the multi-component nature of insect meal and the specific antioxidant and anti-inflammatory properties of astaxanthin will complement or exclude each other. In the groups where lymphocytes levels were higher than in the other groups, the pigs showed no signs of disease and the rearing parameters remained within normal limits. Similarly [30], it was found that the inclusion of *H. illucens* meal in the diet did not significantly affect the blood and serum indices in pigs, but there was an increase in the number of monocytes and neutrophils as the level of this additive increased. Unexpected in our study was the reduction in hemoglobin level in pigs treated with 5% HI larvae meal. Similarly, in the case of serum iron concentration, the addition of HI meal at both levels resulted in a decrease in this parameter. From a physiological point of view, this is detrimental to the body, as the lower the hemoglobin concentration, the worse the circulation of oxygen in the body, and thus the worse the performance of the animal [45]. The lower serum iron levels in the groups with HI larvae meal only were reflected in the red blood cell distribution width (RDWC; *p* < 0.01) and mean corpuscular hemoglobin (MCH; *p* < 0.01). These results contrast with those [45] that showed that replacing 25%, 50%, 75%, or 100% of fish meal with HI meal did not worsen hematological blood parameters, and RBC, HGB, HCT, and RDW were even higher (however, *p* > 0.05) in groups supplemented with HI meal when compared to the control group. In their experiments, HI meal supplementation significantly decreased the platelets counts, while in the present experiment, this parameter was not affected. The lipid fraction of *Hermetia illucens* larvae contains lauric acid in the amount of about 38.43% by weight [46]. It belongs to the saturated fatty acids that exacerbate dyslipidemia, and it is lauric acid that raises circulating cholesterol levels contributing to cardiovascular disease [47]. In our experiment, the supplementation of feed with 2.5 or 5% HI meal (36.5 g of lauric acid per 100 g of all estimated acids) did not influence the cholesterol content in the blood. In contrast, in the experiment by van Heugten et al. (2022) [34] where the HI larvae oil (36.5–37.3 g of lauric acid/100 g) was used in the amount of 2%, 4%, or 6% in the feed, the increase in total cholesterol level (by about 17% compared to control group) was the only significant effect observed in piglets’ biochemical blood indices. These authors, however, did not notice any effect of lauric acid present in HI oil on the hematological parameters.

One mechanism of cardiovascular disease is erythosis. Some studies have confirmed that lauric acid in human red blood cells stimulates erythosis [47]. In addition, the mechanism that affects erythosis is oxidative stress [48], and this stress, according to the above study, is triggered by lauric acid [47]. Hence, it can be assumed that in the present experiment, exposure to lauric acid, in the form of supplementation of *H. illucens* meal, resulted in a decrease in the level of selected red cell parameters. Analyzing further results, a beneficial effect of astaxanthin on these parameters (RBC, Fe, HCT, RDWC) is noticeable. Thus, it can be thought that astaxanthin partially prevents excessive oxidative stress contributing to erythosis. The beneficial effect on limiting oxidative stress was confirmed in studies [49] on broiler chickens receiving from 20 to 80 mg/kg of AST, in which increased catalase and superoxide dismutase levels were observed in plasma. Biochemical blood indices were studied by Yu et al. (2020) [36] on weaned piglets receiving 0%, 1%, 2%, or 4% HI meal in a feed. These authors observed that 2% HI meal increased total protein, IL-10, and IgA while decreasing urea and triglyceride concentration. In the present experiment, the concentration of these biochemical indices was not affected by the HI meal supplementation in feed.

### 4.3. Meat and Backfat Analysis

In the conducted experiment, a significantly higher TBARS value for meat (*longissimus m*.) was noted in the groups receiving astaxanthin, and no effect of HI meal was noticed after storing at −20 °C for 3 months. On the other hand, the astaxanthin added to the feed mixture significantly decreased the TBARS value in adipose tissue (backfat) stored in the same conditions. A significant interaction between the experimental factors was also noted: the highest TBARS value for backfat was in the control group, while the most effective combination of dietary supplements for lowering the TBARS was 2.5% of HI meal together with the astaxanthin. The efficiency of these supplements in improving the shelf life of pork fat was about 80% (2.5% HI meal group) and 77% (AST group and AST + 2.5% HI meal) when compared to the control group. TBARS, expressed as malondialdehyde, is a valuable index of lipid peroxidation and oxidative susceptibility. It reflects the degree of oxidation: the higher the TBARS value, the more intensive oxidation of lipids appears. The beneficial effect of astaxanthin was observed in another study [50] when *longissimus m.* chops originated from the astaxanthin-supplemented pigs had TBARS values more than 60% lower than chops from control pigs after 7 days of retail exposure. Improvement in meat quality was also noticed [49] in broiler chickens fed with 20, 40, or 80 mg/kg of AST, which developed the antioxidant status in breast meat, reduced malondialdehyde levels, and increased redness and yellowness of meat. These results suggest a beneficial effect of AST against lipid oxidation. The results are consistent with the antioxidant activity of AST, which helps protect membrane phospholipids and other lipids from peroxidation [51]. However, some studies [37] did not confirm any significant effects of 1.5 or 3 mg of AST supplementation in the feed for fatteners on the meat TBARS value, drip loss, meat color, and marbling values. This additive was fed to pigs for 14 days only, which could be too short of a period for significant meat quality and oxidative stability affection.

In the present experiment, there was a significantly lower percentage of crude ash in the meat of pigs treated with HI larvae meal. A similar result was obtained in another study where the concentration of ash in breast muscles in broiler chickens (*Pectoralis major*) decreased linearly as the proportion of HI larvae meal in the diet increased [52]. The authors attribute this result to the use of full-fat HI larvae meal, which was also used in our experiment.

## 5. Conclusions

The results of the present study indicate that the inclusion of full-fat meal from *H. illucens* larvae and astaxanthin did not adversely affect feed intake and utilization, daily weight gains, and organ weights in weaned piglets. Both factors, separately and in interaction, have no negative effect on biochemical and hematological blood parameters, which remained within the norms. It seems that astaxanthin supplemented even in small amounts supports the inhibition of oxidative stress, which became apparent in the case of some red blood cell parameters. The 2.5% full-fat *H. illucens* larvae meal and astaxanthin, used in feed mixture separately or together, can reduce the susceptibility of pork fat to the oxidation process and improve its shelf life. It is suggested that the higher concentration of *H. illucens* meal (5%) should not be used, as the presence of lauric acid can cause adverse changes in some of the red cell indices. However, using the HI meal along with the antioxidant astaxanthin improves these indices.

## Figures and Tables

**Table 1:** Ingredients (%) and nutritive value of diets in the experiment.

	I	II	III	IV	V	VI
Items	Control	HI 2.5%	HI 5%	HI 2.5% + AST	HI 5% + AST	AST
Soybean pressed cake	19	16	13	16	13	19
*Hermetia illucens* larvae meal	-	2.5	5	2.5	5	-
Wheat	42.2	43.6	44.2	43.6	44.2	42.2
Corn	20	20	20	20	20	20
Rapeseed oil	0.8	-	-	-	-	0.8
Skimmed milk powder	10	10	10	10	10	10
Dried whey	5	5	5	5	5	5
1-Ca phosphate	0.4	0.3	0.3	0.3	0.3	0.4
Feed chalk	1.2	1.1	1.1	1.2	1.1	1.2
Salt	0.1	0.1	0.1	0.1	0.1	0.1
Lysine	0.4	0.4	0.4	0.4	0.4	0.4
Methionine	0.2	0.2	0.2	0.2	0.2	0.2
Threonine	0.2	0.2	0.2	0.2	0.2	0.2
Tryptophan	0.1	0.1	0.1	0.1	0.1	0.1
Vitamin–mineral premix *	0.5	0.5	0.5	0.5	0.5	0.5
Astaxanthin (0.025 g/kg)	-	-	-	+	+	+
Content in 1 kg:						
Dry matter, g	901	899	898	899	898	901
Crude protein, g	183	183	183	183	183	183
Crude fat, g	40	36	40	36	40	40
Crude fiber, g	27	28	29	28	29	27
Crude ash, g	56	55	54	55	54	56
Metabolizable energy, MJ **	13.8	13.7	13.7	13.7	13.7	13.8
Lysine, g	13.7	13.7	13.6	13.7	13.6	13.7
Methionine + Cystine, g	8.2	8.2	8.2	8.2	8.2	8.2
Threonine, g	8.8	8.8	8.7	8.8	8.7	8.8
Tryptophan, g	2.7	2.7	2.7	2.7	2.7	2.7
Calcium, g	7.6	7.7	7.6	7.7	7.6	7.6
Phosphorus digestible, g	3.1	3.1	3.1	3.1	3.1	3.1

Abbreviations: *HI—Hermetia illucens larvae meal*; *AST—astaxanthin.* I, II, III, IV, V, VI—number of groups: group I—control, group II—addition of 2.5% *Hermetia illucens* (HI) larvae meal, group III—addition of 5% *H. illucens* larvae meal, group IV—addition of 2.5% *H. illucens* larvae meal and astaxanthin, group V—addition of 5% *H. illucens* larvae meal and astaxanthin, group VI—addition of astaxanthin. * Content in 1 kg of premix: vit A—2,400,000 IU; vit D3—400,000 IU; vit E—8000 IU; vit B1—400 mg; vit B12—6000 µg; vit B2—1000 mg; vit B5—3000 mg; vit B6—600 mg; vit K—400 mg; biotin—30,000 µg; niacin—5008.3 mg; folic acid—100 mg; pantothenic acid—2760 mg; choline—24,193.548 mg; betaine—12,000 mg; Cu—20,000 mg; Fe—20,000 mg; I—200 mg; Mn—8000 mg; Se—60 mg; Zn—24,000 mg; Ca—267.979 g; Cl—6.268 g; K—0.066 g; Mg—30 g; Na—0.037 g; S—22.245 g. ** Metabolizable energy was calculated using Hoffmann and Schiemann’s equation (1980) [25].

**Table 2:** Effect of *Hermetia illucens* meal share and astaxanthin presence in feed on the productivity performance of piglets.

	*Hermetia illucens* Share (HI)			Astaxanthin Share (AST)		I	II	III	IV	V	VI	*p*-Level			
Items	0	2.5	5	-	+	Control	HI 2.5%	HI 5%	HI 2.5% + AST	HI 5% + AST	AST	HI	AST	HI × AST	SEM
Average body weight, kg															
1st day of experiment	8.74	8.64	8.75	8.70	8.72	8.89	8.58	8.64	8.70	8.85	8.60	0.886	0.941	0.578	0.10
35th day of experiment	34.19	34.46	34.19	34.28	34.29	34.36	34.43	34.05	34.49	34.32	34.05	0.883	0.988	0.897	0.24
Average daily weight gains, g															
1–35 day of experiment	727	738	727	731	730	728	738	726	737	728	727	0.692	0.985	0.992	5.63
Average daily feed intake, kg															
1–35 day of experiment	1.08	1.08	1.08	1.08	1.08	1.08	1.09	1.08	1.08	1.08	1.08	0.959	0.991	0.781	0.01
Feed conversion ratio, kg															
1–35 day of experiment	1.49	1.47	1.50	1.48	1.48	1.49	1.46	1.50	1.47	1.49	1.49	0.191	0.876	0.853	0.01

I, II, III, IV, V, VI—number of groups; *Hermetia illucens* share of 0% (groups I and VI), *Hermetia illucens* share of 2.5% (group II and group IV), *Hermetia illucens* share of 5% (group III and group V) and astaxanthin supplementation (groups IV, V, and VI), without astaxanthin supplementation (groups I, II, and III).

**Table 3:** Effect of *Hermetia illucens* meal share and astaxanthin presence in feed on the weight of organs and digestive tract sections of piglets.

	*Hermetia illucens* Share (HI)			Astaxanthin Share (AST)		I	II	III	IV	V	VI	*p*-Level			
Items	0	2.5	5	-	+	Control	HI 2.5%	HI 5%	HI 2.5% + AST	HI 5% + AST	AST	HI	AST	HI × AST	SEM
Liver	2.63	2.63	2.62	2.61	2.64	2.63	2.60	2.61	2.65	2.62	2.64	0.96	0.665	0.953	0.03
Spleen	0.21	0.20	0.20	0.21	0.21	0.21	0.21	0.20	0.20	0.20	0.21	0.522	0.917	0.972	0.003
Kidney	0.27	0.28	0.27	0.28	0.27	0.28	0.28	0.27	0.27	0.28	0.27	0.977	0.687	0.797	0.004
Stomach	0.74	0.75	0.74	0.75	0.74	0.74	0.75	0.74	0.75	0.73	0.73	0.908	0.596	0.963	0.01
Duodenum	0.08	0.07	0.08	0.08	0.08	0.08	0.07	0.08	0.07	0.08	0.08	0.262	0.513	0.34	0.002
Caecum	0.28	0.27	0.28	0.28	0.27	0.27	0.28	0.27	0.27	0.28	0.28	0.965	0.886	0.742	0.003
Small intestine	3.37	3.37	3.35	3.35	3.37	3.35	3.38	3.33	3.35	3.36	3.39	0.967	0.862	0.907	0.04
Large intestine	1.67	1.69	1.66	1.67	1.68	1.68	1.69	1.65	1.70	1.66	1.67	0.746	0.901	0.987	0.02

I, II, III, IV, V, VI—number of groups; *Hermetia illucens* share of 0% (groups I and VI), *Hermetia illucens* share of 2.5% (group II and group IV), *Hermetia illucens* share of 5% (group III and group V) and astaxanthin supplementation (groups IV, V, and VI), without astaxanthin supplementation (groups I, II, and III).

**Table 4:** Effect of *Hermetia illucens* meal share and astaxanthin presence in feed on the biochemical indices of piglets’ blood.

	*Hermetia illucens* Share (HI)			Astaxanthin Share (AST)		I	II	III	IV	V	VI	*p*-Level			
Items	0	2.5	5	-	+	Control	HI 2.5%	HI 5%	HI 2.5% + AST	HI 5% + AST	AST	HI	AST	HI × AST	SEM
Lipid profile															
CHOL, mg/dL	97.4	100.4	93.7	96.0	98.4	95.7	102.8	89.8	98.1	97.6	99.4	0.415	0.573	0.458	2.01
TG, mg/dL	41.0	46.2	36.8	42.5	40.1	43.1	46.4	38.0	46.0	35.6	38.6	0.063	0.445	0.870	1.60
HDL, mg/dL	40.5 ^ab^	44.7 ^a^	38.7 ^b^	40.3	42.2	38.8	44.7	37.6	44.6	39.8	42.3	0.031	0.321	0.716	0.94
LDL, mg/dL	51.3	49.4	49.1	50.4	49.5	51.7	51.8	47.6	46.9	50.6	50.8	0.767	0.736	0.487	1.28
LDH, U/L	1324.7 ^a^	1482.1 ^a^	1706.8 ^b^	1528	1472.5	1300.7	1556.6	1755.1	1407.5	1658.4	1351.7	<0.01	0.426	0.576	45.10
Hepatic and pancreas profile															
ALT, U/L	46.4	48.7	46.6	48.0	46.5	46.7	51.4	46.0	46.0	47.3	46.2	0.756	0.570	0.590	1.32
AST, U/L	50.3	57.1	54.9	55.9	52.2	50.4	58.6	59.3	55.6	50.6	50.2	0.483	0.406	0.758	2.28
ALP, U/L	237.5	255.4	261.4	234.8 ^a^	268.2 ^b^	263.4 ^ab^	217.5 ^a^	220.0 ^a^	293.4 ^b^	302.8 ^b^	208.4 ^a^	0.374	0.031	<0.01	9.08
GLU, mg/dL	135.6 ^a^	136.4 ^a^	120.9 ^b^	121.2 ^a^	141.3 ^b^	131.8	124.1	106.3	148.6	135.5	139.9	0.046	<0.01	0.269	3.25
ALB, g/dL	4.2	3.9	3.9	3.9	4.1	4.34 ^d^	3.8 ^ab^	3.7 ^a^	4.1 ^bcd^	4.2 ^cd^	4.0 ^abc^	0.058	0.115	<0.01	0.05
Renal profile															
CREA, mg/dL	1.0 ^a^	0.9 ^b^	1.0 ^ab^	0.9 ^a^	1.0 ^b^	1.0	0.8	0.9	1.0	1.1	1.0	0.022	<0.01	0.177	0.02
UREA, mg/dL	23.8	21.3	22.4	22.2	22.8	25.8	20.3	20.2	22.3	24.5	21.6	0.591	0.712	0.187	0.96
TP, g/dL	6.3	6.2	6.0	6.1	6.3	6.4 ^b^	6.1 ^b^	5.6 ^a^	6.3 ^b^	6.4 ^b^	6.2 ^b^	0.174	0.051	<0.01	0.07
Ca, mg/dL	12.2 ^a^	12.7 ^a^	10.9 ^b^	11.5 ^a^	12.4 ^b^	11.8	12.6	10.0	12.9	11.8	12.6	<0.01	<0.01	0.085	0.19
P, mg/dL	10.3 ^a^	11.4 ^b^	9.6	10.6	10.3	10.5	11.3	10.0	11.4	9.2	10.1	<0.01	0.322	0.581	0.19
Mg, mg/dL	2.2	2.3	2.2	2.1 ^a^	2.4 ^b^	2.1	2.3	2.0	2.4	2.3	2.3	0.162	<0.01	0.603	0.05
Osteological profile															
Ca, mg/dL	12.2 ^a^	12.7 ^a^	10.9 ^b^	11.5 ^a^	12.4 ^b^	11.8	12.6	10.0	12.9	11.8	12.6	<0.01	<0.01	0.085	0.19
P, mg/dL	10.3 ^a^	11.4 ^b^	9.6 ^a^	10.6	10.3	10.5	11.3	10.0	11.4	9.2	10.1	<0.01	0.322	0.581	0.19
ALP, U/L	237.5	255.4	261.4	234.8 ^a^	268.2 ^b^	263.4 ^ab^	217.5 ^a^	220.0 ^a^	293.4 ^b^	302.8 ^b^	208.4 ^a^	0.374	0.031	<0.01	9.08
ALB, g/dL	4.2	3.9	3.9	3.9	4.1	4.3 ^d^	3.8 ^ab^	3.6 ^a^	4.1 ^bcd^	4.2 ^cd^	3.9 ^abc^	0.058	0.115	<0.01	0.05

I, II, III, IV, V, VI—number of groups; *Hermetia illucens* share of 0% (groups I and VI), *Hermetia illucens* share of 2.5% (group II and group IV), *Hermetia illucens* share of 5% (group III and group V) and astaxanthin supplementation (groups IV, V, and VI), without astaxanthin supplementation (groups I, II, and III).^a,b,c,d^—values within a row with different superscripts differ significantly at *p* ≤ 0.05. Abbreviations: CHOL—total cholesterol, HDL—high-density lipoprotein, LDL—low-density lipoprotein, TG—triacylglycerides, LDH—lactate dehydrogenase, ALT—alanine aminotransferase, AST—aspartate aminotransferase, ALP—alkaline phosphatase, GLU—glucose, ALB—albumin, CREA—creatinine, UREA—urea, TP—total protein, Ca—calcium, P—phosphate, Mg—magnesium.

**Table 5:** Effect of *Hermetia illucens* meal share and astaxanthin presence in feed on the hematological indices of piglets’ blood.

	*Hermetia illucens* Share (HI)			Astaxanthin Share (AST)		I	II	III	IV	V	VI	*p*-Level			
Items	0	2.5	5	-	+	Control	HI 2.5%	HI 5%	HI 2.5% + AST	HI 5% + AST	AST	HI	AST	HI × AST	SEM
WBC, 10^3^/μL	9.5	11.4	12.0	10.4	12.2	9.9	10.6	10.6	12.3	14.8	8.5	0.093	0.201	0.203	0.57
LYM, 10^3^/μL	4.5 ^a^	6.3 ^ab^	7.0 ^b^	5.3	7.2	4.4	5.1	6.3	7.6	8.4	4.6	0.043	0.058	0.508	0.43
MON, 10^3^/μL	0.5	0.4	0.4	0.4	0.4	0.4 ^ab^	0.5 ^ab^	0.4 ^a^	0.3 ^a^	0.6 ^b^	0.5 ^ab^	0.444	0.308	0.012	0.02
GRA, 10^3^/μL	4.6	4.7	4.5	4.6	4.6	5.1 ^ab^	5.0 ^ab^	3.9 ^ab^	4.4 ^ab^	5.8 ^b^	3.4 ^a^	0.657	0.733	0.022	0.24
RBC, 10^6^/μL	6.2	6.5	6.1	5.7 ^a^	6.9 ^b^	5.7	6.1	5.4	6.9	6.9	6.8	0.210	<0.01	0.218	0.12
HGB, g/dL	12.9 ^a^	12.7 ^a^	11.7 ^b^	13.0 ^a^	11.8 ^b^	13.9	13.4	11.8	12.0	11.7	11.8	0.024	<0.01	0.081	0.20
Fe, µg/dL	140.3 ^a^	116.4 ^b^	107.0 ^b^	106.1 ^a^	137.8 ^b^	125.8	104.1	86.0	128.8	128.0	156.6	0.011	<0.01	0.743	5.37
HCT %	39.8 ^a^	41.0 ^a^	35.6 ^b^	35.5 ^a^	42.3 ^b^	37.7 ^b^	38.3 ^bc^	30.3 ^a^	43.7 ^d^	41.0 ^cd^	42.2 ^d^	<0.01	<0.01	<0.01	0.74
RDWC %	18.8 ^a^	18.0 ^a^	20.0 ^b^	18.5 ^a^	19.4 ^b^	18.5	17.2	19.8	18.7	20.2	19.2	<0.01	0.032	0.518	0.23
MCV, μm^3^	64.0 ^a^	63.4 ^a^	58.4 ^b^	62.2	61.7	65.7 ^d^	63.2 ^cd^	57.1 ^a^	63.5 ^cd^	59.6 ^ab^	62.1 ^bc^	<0.01	0.724	0.025	0.59
MCH, pg	21.3 ^a^	19.8 ^b^	19.6 ^b^	22.9 ^a^	17.5 ^b^	24.2	22.1	22.3	17.5	17.0	18.0	<0.01	<0.01	0.120	0.43
PLT, 10^3^/μL	220.4	272.6	235.8	241.8	243.2	211.4	277.4	240.3	267.8	231.3	230.5	0.068	0.993	0.760	9.19
PDW %	39.9 ^a^	31.9 ^b^	43.6 ^a^	36.9	40.2	37.3	31.2	42.0	32.5	45.2	42.9	<0.01	0.079	0.652	1.16
PCT %	0.1	0.2	0.2	0.2	0.1	0.1	0.2	0.2	0.2	0.1	0.1	0.127	0.967	0.605	0.01
MPV, μm^3^	8.3	8.4	8.8	8.3	8.7	8.1	8.2	8.7	8.7	8.9	8.4	0.055	0.076	0.838	0.09

I, II, III, IV, V, VI—number of groups; *Hermetia illucens* share of 0% (groups I and VI), *Hermetia illucens* share of 2.5% (group II and group IV), *Hermetia illucens* share of 5% (group III and group V) and astaxanthin supplementation (groups IV, V and VI), without astaxanthin supplementation (groups I, II and III). ^a,b,c,d^—values within a row with different superscripts differ significantly at *p* ≤ 0.05. Abbreviations: WBC—white blood cells; LYM—lymphocytes; MON—monocytes; GRA—granulocytes; RBC—red blood cells; HGB—hemoglobin; Fe—iron; HCT—hematocrit; RDWC—red blood cell distribution width; MCV—mean corpuscular volume; MCH—mean corpuscular hemoglobin; PLT—platelets; PDW—platelet distribution width; PCT—plateletcrit; MPV—mean platelet volume.

**Table 6:** Effect of *Hermetia illucens* meal share and astaxanthin presence in feed on the basic chemical analyses of meat (*longissimus m*.) and the oxidative stability of meat and backfat tissue.

	*Hermetia illucens* Share (HI)			Astaxanthin Share (AST)		I	II	III	IV	V	VI	*p*-Level			
Items	0	2.5	5	-	+	control	HI 2.5%	HI 5%	HI 2.5% + AST	HI 5% + AST	AST	HI	AST	HI × AST	SEM
Nutrient content in meat															
Dry matter (DM), %	23.5 ^a^	24.1 ^b^	23.6 ^a^	23.8	23.7	23.2 ^a^	24.2 ^c^	23.9 ^bc^	23.9 ^bc^	23.4 ^ab^	23.8 ^bc^	0.007	0.706	0.016	0.086
Protein, % in DM	87.8	80.4	86.2	82.9	86.9	88.3	74.4	85.2	86.3	87.2	87.1	0.074	0.123	0.132	1.445
Fat, % in DM	7.7	7.5	7.3	7.9	7.1	8.4	7.6	7.5	7.4	7.2	6.8	0.871	0.112	0.348	0.214
Ash, % in DM	5.2 ^a^	4.3 ^b^	5.0 ^a^	4.7 ^a^	5.0 ^b^	5.1	4.1	4.9	4.6	5.1	5.3	<0.010	0.030	0.633	0.009
TBARS content in tissues (mg/kg)															
Meat (*longissimus m.*)	0.3	0.3	0.3	0.3 ^a^	0.3 ^b^	0.3	0.3	0.2	0.3	0.3	0.3	0.160	0.002	0.144	0.010
Adipose tissue (backfat)	0.8 ^a^	0.3 ^b^	0.4 ^b^	0.7 ^a^	0.3 ^b^	1.3 ^a^	0.3 ^b^	0.4 ^b^	0.3 ^b^	0.4 ^b^	0.3 ^b^	<0.010	<0.010	<0.010	0.068

I, II, III, IV, V, VI—number of groups; *Hermetia illucens* share of 0% (groups I and VI), *Hermetia illucens* share of 2.5% (group II and group IV), *Hermetia illucens* share of 5% (group III and group V) and astaxanthin supplementation (groups IV, V, and VI), without astaxanthin supplementation (groups I, II, and III). ^a,b,c^—values within a row with different superscripts differ significantly at *p* ≤ 0.05.

## Data Availability

The data supporting reported results are in the possession of the authors (K.S. and M.Ś.)
